# An Old Physician’s Advice

**Published:** 1879-08

**Authors:** W. W. Hall


### *AN OLD PHYSICIAN'S ADVICE.*

By W. W. Hall, A. M., M. D.

*Founder of the "Journal of Health."*

Forty years of active and unremitting service as a physician and writer, with the study and investigation requisite to whatever success I may have achieved, long ago convinced me that a majority of the diseases that afflict us and shorten our lives have their origin in derangements of the liver, and that they are nearly all preventable or curable by proper treatment.

I shall endeavor to concentrate in this article a few of the most essential facts that may be found scattered through the pages of the Journal during the more than twenty years of its existence—facts and rules relating to the preservation of health of more value to ourselves and our children than riches. Yet not one in five, who read this article, will remember for one day a single fact stated, or, remembering, will heed it. Hence the great mass of mankind perishes before its prime. And right here I will make an explanation, which is something I never remember to have done before, in regard to my professional course.

Some may wonder that I should advise the use of "advertised remedies." I have employed a few carefully selected remedies of this description, because I have found them more reliable than any others, and for no other reason. I have no pecuniary interest whatever in any of them. It would be unwise to reject good medicines because people are asked to try them. In my practice I employ but few drugs, and these cautiously. I use them as servants to do my bidding, but I have never permitted them to get the mastery over me. Like fire, they are useful servants, but dangerous masters. A single dose of some active drug, *wisely prescribed*, would often save a valuable life.

I will now describe briefly some of the diseases that are often neglected until they sap the foundations of life, or by improper treatment quickly destroy it:

